# Exploring the effects of negative supervisory feedback on creativity among research and development personnel: challenge or threat?

**DOI:** 10.3389/fpsyg.2024.1361616

**Published:** 2024-07-03

**Authors:** Haihong Li, Jianwei Zhang, Muhammad Yaseen Bhutto, Myriam Ertz, Jie Zhou, Xingyu Xuan

**Affiliations:** ^1^School of Business Administration, Shandong University of Finance and Economics, Jinan, China; ^2^School of Education, Beijing Institute of Technology, Beijing, China; ^3^Business School, Shandong Jianzhu University, Jinan, China; ^4^LaboNFC, Department of Economics and Administrative Sciences, University of Quebec in Chicoutimi, Saguenay, QC, Canada; ^5^Department of Police Management, Sichuan Police College, Luzhou, China; ^6^School of Public Administration, Shanxi University of Finance and Economics, Taiyuan, China

**Keywords:** negative supervisory feedback, challenge appraisal, threat appraisal, prevention focus, employee creativity

## Abstract

Supervisory feedback to stimulate research and development (R&D) employee creativity is a management issue that concerns scholars and practitioners. However, there are divergences and contradictions regarding whether negative feedback promotes or hinders employee creativity. Integrating the feedback intervention and cognitive appraisal theories, we developed a double-edged sword model for negative supervisory feedback's influence on creativity. We tested the proposed model using a field sample of 513 R&D employees from seven science and technology enterprises. The results indicated that R&D employee challenge and threat appraisal moderated negative supervisory feedback's effect on prevention focus and the distal consequences for creativity. Individuals with high (low) levels of challenge (threat) appraisal have decreased prevention focus, thereby increasing their creativity when receiving negative supervisory feedback. In contrast, individuals with low (high) challenge (threat) appraisal have increased prevention focus, thereby decreasing their creativity when receiving negative supervisory feedback. These findings offer interesting implications for research on negative feedback and stimulation of science and technology R&D employee creativity in organizations.

## 1 Introduction

Employee creativity—defined as the production of novel and useful ideas (Amabile, 1988)—is an essential source of sustained innovation and gives organizations a competitive advantage. It allows organizations to continually produce innovative and creative products, services, and processes. Research and development (R&D) personnel is the leading force in driving enterprises to combine customer needs with new industrial practices and technology potentials to create new products, processes and technologies (Tang et al., 2014). In this context it is of great interest to understand the topic of how to stimulate R&D employee creativity. Managers often view negative feedback as a positive tool to motivate subordinates' creative behaviors and achieve performance improvement (Zenger and Folkman, 2017). This is because it points directly to where an individual's current creativity problem lies, creating awareness of a gap between the current creativity and the norm (Kim and Kim, 2020). However, there is little consensus on the direction and contingencies of negative feedback's effect on employee creativity. Some scholars suggest that negative feedback does not directly affect creativity (Hon et al., 2013), and others argue that it can inhibits employee creativity (Van Dijk and Kluger, 2011; Liu et al., 2022). Meanwhile, some research findings indicate that negative feedback is actually positively associated to the recipient's creativity and innovation (Hoever et al., 2018; Ma et al., 2021). Such perplexing empirical evidence indicates that a fundamental question remains unanswered—how does negative supervisory feedback influence employee creativity and why?

Feedback intervention theory provides a theoretical perspective to explain the link between negative supervisory feedback and employee creativity (Kluger and DeNisi, 1996; Dahling et al., 2016). Feedback intervention theory suggests that negative feedback makes feedback recipients aware of a gap between their current level of creativity and the standards, and this awareness leads them to generate two functionally opposite tracks of attention in responding to the negative feedback: task-processes and meta-processes. Negative feedback's effectiveness decreases as attention moves closer to the meta-processes and away from the task-processes. On the one hand, when feedback recipients focus their attention on the task-process, negative feedback activates recipients' task-based reflection and learning, prompting them to constructively improve and close the above gap by trying to design and implement more novel and valuable strategies (Dong et al., 2023). On the other hand, when feedback recipients focus their attention on meta-processes, the discrepancy between feedback and norm makes recipients feel more threatened concerning their self-concept, generating a prevention focus motivation (Brockner and Higgins, 2001). This in turn hinders their creative efforts and attempts (Kim and Kim, 2020).

Predictions based on feedback intervention theory's perspective explain the inconsistent findings between negative feedback and creativity and raise an essential theoretical and practical question: when do employees receiving negative feedback from their leaders focus on task-processes to positively affect their creativity? When does a focus on meta-processes negatively affect their creativity? We research this question to determine whether the answer depends on a critical dispositional characteristic—cognitive appraisal (including challenge and threat appraisal)—which influences how negative feedback recipients' attention changes during feedback-standard comparison. Conducting a field study, we find that a recipient's cognitive appraisal determines whether receiving negative supervisory feedback harms or helps their own prevention focus and creativity. The cognitive appraisal theory of stress emphasizes that stress appraisal and stress coping play an essential role in the relationship between stressors and outcomes, and different stress appraisal styles lead to diverse coping processes. Challenge appraisal focuses on potential growth, mastery, or gain, whereas threat appraisal focuses on potential harm or loss (Folkman and Lazarus, 1985; LePine et al., 2016). Although an appraisal can be an assessment of a specific stressful event, individuals actually have different general tendencies to appraisal stressful events before they occur (Skinner and Brewer, 2002). In view of this, recent research calls for scholars to explore the moderating effect of stress cognitive appraisal (O'Brien and Beehr, 2019). Therefore, we believe that negative feedback's effect differs significantly according to individual cognitive appraisal styles. Specifically, when individuals make more challenge appraisals, they are more likely to focus on the task level, actively seek improvement strategies, and increase creative engagement to gain growth opportunities. In this case, negative feedback is positively related to creativity through a prevention focus. When individuals make more threat appraisals, they are more likely to focus on the injuries or losses at the self-level, which generates prevention mechanisms and weakens creative efforts. In this case, negative feedback was negatively related to creativity through prevention focus.

**Figure 1:**
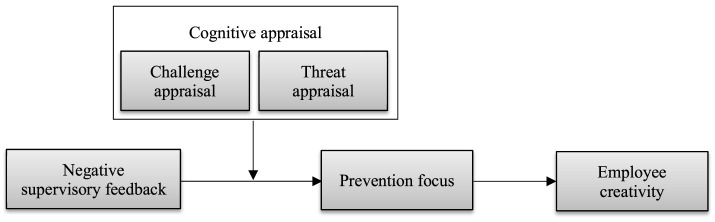
Theoretical model.

Integrating feedback intervention and cognitive appraisal theories highlights recipients' responses to negative feedback from supervisors, contributing to several relevant literature streams. First, it integrates contradictory findings on the relationship between negative feedback and creativity. In contrast to the single relationship found in previous theoretical and empirical studies, we believe the relationship between negative feedback and creativity can be both negative and positive, depending on individual differences in feedback recipients. Because facing with the same work stressor, individuals' varying appraisal tendencies can influence their selection of stress coping processes, ultimately resulting in different creative results (Dong et al., 2024). This finding addresses the inconsistency of the relationship between negative feedback and creativity and responds to scholars' calls to examine the boundary conditions between the two (Dong et al., 2023). Second, our study expands the boundary conditions of the relationship between negative feedback and motivation. By integrating cognitive appraisal and feedback intervention theories, we argue that negative feedback recipients would regulate their motivation strategies according to their different cognitive appraisal styles. The above finding proposes a boundary condition between negative feedback and self-regulating motivation from feedback recipients' perspective, which helps deepen the understanding of the relationship between negative feedback and motivational consequences (Li et al., 2014). Finally, our research enriches readers' understanding of the mediating transmission mechanism between negative feedback and creativity. Based on the regulatory focus theory, this investigation examines the mediating effect of prevention focus—a more explicit motivational concept—between negative feedback and situational variables on creativity. We break through the previous framework of intrinsic motivation (Zhou, 2008), providing a new viewpoint for research in this field. Figure 1 shows the theoretical model.

## 2 Literature review and hypothesis development

### 2.1 Negative supervisory feedback and prevention focus

For creative tasks, supervisory feedback valence refers to positive or negative outcome of the supervisor's comparison between individual's creative performance and situational criteria (Zhou, 1998). This provides leaders with a criterion for evaluating the creativity of employees' work ideas and processes. Positive feedback signals that the individual's ideas or behaviors are more creative than the criteria; negative feedback indicates that the ideas or behaviors are less creative than the criteria (Zhou, 1998). According to Jaworski and Kohli (1991)'s suggestion, the above definition includes two dimensions of feedback—output feedback (e.g., creative ideas) and behavioral feedback (e.g., creative strategies or routines). Because previous research has indicated that a single feedback construct may result in inconsistent findings regarding the relationship between feedback and its outcomes (Jaworski and Kohli, 1991). By highlighting for the discrepancies between employees' performance and goals and motivating them to improve their performance, supervisory feedback can help adjust and regulate employee behaviors (Brendl and Higgins, 1996; Xing et al., 2023). Previous research has examined various incentive effects of supervisory feedback valence such as intrinsic motivation, performance results, and creativity. These studies found that positive feedback is usually related to approach behaviors, such as higher levels of creativity (Hoever et al., 2018) and task performance (Su and Jiang, 2023). In contrast, negative feedback is usually related to avoidance behaviors, such as avoiding potential losses (Li et al., 2014). Although the above findings provide empirical evidence of relationship between feedback valence and individual motivation, they offer little knowledge of the specific motivational patterns the two types of feedback valence trigger on employee behavior. Incorporating regulatory focus theory into the feedback valence literature to study its motivational consequences helps address this gap (Li et al., 2014).

In recent years, regulatory focus theory has contributed a new perspective to human motivation research. It focuses on how individuals approach the positive target state and avoid the negative one. Higgins (1997) argued that there are two different motivation systems for self-regulation in the process of individuals approaching happiness and avoiding pain. The author also proposed that there are two motivations related to the needs for self-realization and security, ideal-self and realistic-self, namely, promotion focus, and prevention focus, revealing two self-regulation motivation modes of individual behavior. Promotion focus reflects the needs of an individual's growth, improvement, and development, positioning an individual as an ideal self, narrowing the gap between realistic and ideal states through self-regulation, and experiencing work as obtaining or not obtaining. Prevention focus reflects the needs for individual security and stability, positioning the individual in the ought self, narrowing the gap between reality and the ideal state through self-regulation, and experiencing work as loss or no loss. There are two states of regulatory focus: long-term and situational. The former is a relatively stable individual difference that individual personality traits influence. The latter is a relatively short and immediate state that environmental elements activate (Scholer et al., 2019). In this study, prevention focus was defined as the latter. As a prominent environmental cue in the workplace, leader behavioral patterns have an essential impact on arousing employee promotion or prevention focus (Brockner et al., 2004; Kark et al., 2018). Many studies have focused on the relationship between leadership (e.g., supervisory feedback valence) and employee regulatory focus in organizations (Kark and Van Dijk, 2019).

A review of previous studies showed that negative supervisory feedback positively relates to an individual's prevention focus. Supervisory feedback valence has an indicative function because it enables employees to obtain information about their progress toward achieving their goals, thus enabling them to adjust their performance strategies accordingly (Locke and Latham, 2002). Failure information conveys a negative performance signal, resulting in denial of and opposition to the employees' performance and ability, and causing negative emotions such as vigilance and nervousness, which tend to choose avoidance strategies (Idson et al., 2000). When a leader uses negative feedback in response to subordinates' failure, it motivates them to focus on safety, obligations, and responsibility to prevent committing mistakes, that is, triggering subordinates' prevention focus (Brockner and Higgins, 2001). According regulatory focus theory, the external environment influences regulatory focus, mainly situational regulatory focus. A work environment that pays attention to security and transmits information about loss will quickly lead to employees' prevention focus (Scholer et al., 2019). This is because linguistic frameworks that emphasize security and loss stimulate employees' internal need for security and stability, awaken their personal position of the ought self, and form a situational atmosphere that emphasizes loss or no loss. As a result, employees tend to adopt conservative and vigilant strategies to deal with their work, and through self-regulation, their work performance reaches the ought state of their duties and obligations to minimize “loopholes” and “uncertainties” and avoid losses and mistakes. Recent studies provided empirical evidence for this argument. They found that when leaders offer negative feedback to subordinates, emphasizing responsibilities, obligations, and duties, subordinates' prevention focus will be activated (Li et al., 2014; Su and Zhang, 2023). However, the above positive association between negative supervisory feedback and prevention focus does not provide a comprehensive understanding of the two, because studies have largely ignored individual difference factors (Ma et al., 2021; Dong et al., 2023). Individuals with different traits receiving negative supervisory feedback may produce differentiated motivational and behavioral responses. To prove this point, the present study introduces cognitive appraisal as the main individual difference variable based on feedback intervention and cognitive appraisal theories. It examines challenge appraisal's and threaten appraisal's differential moderating effects in the relationship between negative supervisory feedback and prevention focus and its consequences for creativity, respectively. The study's aim is to offer a more detailed explanation of negative supervisory feedback's consequences.

### 2.2 Moderating effect of challenge appraisal and threat appraisal

Based on the cognitive appraisal theory of stress, scholars have found that individual cognitive appraisal provides an important perspective for explaining workplace stressors' positive and negative effects (Kong et al., 2023). Lazarus and Folkman (1984) suggested that cognitive appraisal can be divided into two processes: initial appraisal and reappraisal. The first appraisal assesses stressful situations' gain and loss potential, and the second evaluates an individual's ability to cope with such situations. The initial evaluation process produces two appraisal styles: challenge appraisal and threat appraisal. Challenge appraisal focuses on potential growth, control, or gain in a stressful situation, whereas threat appraisal focuses on possible injuries or losses (Folkman and Lazarus, 1985; LePine et al., 2016). Consistent with Lazarus and Folkman (1984)'s theory, Webster et al. (2011) showed that the two cognitive appraisal styles are not mutually exclusive, and that the same stress situation in the workplace can be evaluated as both a challenge and a threat. Previous studies have found that cognitive appraisal can affect work outcomes by influencing employee intrinsic motivations, emotions, and coping strategies. Challenge appraisal can enhance intrinsic motivation and increase positive emotions and problem-focused coping strategies, whereas threat appraisal can reduce intrinsic motivation and increase negative emotions and emotion-focused coping strategies (LePine et al., 2005; Searle and Auton, 2015).

According to feedback intervention theory, feedback's effectiveness depends on t feedback recipients' individual attributes; that is, whether negative feedback recipients focus their attention on the task or self-level (Kluger and DeNisi, 1996; Dahling et al., 2016). When feedback receivers perceive feedback at the task level, their primary concern is improving task performance to achieve goals or expectations. In contrast, when they perceive feedback at the self-level, a defense mechanism is generated, leading to a decrease in effort and performance. The cognitive appraisal theory of stress points out that a work situation characterized by social evaluation constitutes employees' psychological stressor (Byron et al., 2010). Negative supervisory feedback, a negative performance indicator, is a typical psychological stressor. Employee cognitive appraisal of this stressor determines whether they focus on the task- or self-level after receiving feedback. In other words, challenge appraisal and threat appraisal moderate the relationship between negative supervisory feedback and employee prevention focus.

Specifically, challenge appraisal refers to an individual's inclination to realize potential benefits or opportunities (Gutnick et al., 2012; Mitchell et al., 2019). When the challenge appraisal level is high, employees tend to focus their attention on the task level, triggering their problem-focused coping strategies (Searle and Auton, 2015; Kong et al., 2023), reflecting on the creative problems and gaps negative supervisory feedback has pointed out; actively seeking performance improvement strategies; and looking for opportunities for growth, promotion, and development. Additionally, challenge appraisal generates employee approach goal orientation, prompting employees to attempt to narrow the difference between current and expected states to reach the ideal standard (Higgins, 1997; Gutnick et al., 2012). Thus, negative performance indication's effect on negative supervisory feedback is less likely to work for challenge appraisal employees who focus on tasks. Behavioral plasticity theory provides evidence for this inference. Scholars believe that people with positive self-evaluations are unlikely to rely on external cues to guide their motivation and behavior, so other people's performance-related perceptions are unlikely to influence them (Brockner, 1988). In contrast, receiving negative feedback may also stimulate employee state promotion focus, strengthen employees' need for growth and development, prompt them to strive to achieve their ideal self, and increase their desire to pursue a sense of gain at work. When the employee challenge appraisal level is low, employees devote less attention and energy to the task level and are less likely to generate problem-focused coping strategies or approach goal orientation. Supervisory negative feedback may not have a negative association with employee prevention focus in this case. Current empirical findings suggest the above reasoning. Li et al. (2017) found that individuals who challenge the appraisal of perceived creative reward as a stressor have higher intrinsic motivation and achieve better creative performance. Accordingly, we propose the following hypothesis:

H1: R&D employee challenge appraisal negatively moderates the relationship between negative supervisory feedback and prevention focus such that the positive relationship is weaker when challenge appraisal is higher than when it is lower.

We also predict the interaction between negative supervisory feedback and threat appraisal on prevention focus. A high threat appraisal level is often associated with expected loss, which reflects a psychological state that tends to avoid adverse outcomes (Gutnick et al., 2012; Mitchell et al., 2019). At this point, employees tend to focus their attention on the self-level, triggering emotion-focused coping strategies (Searle and Auton, 2015; Kong et al., 2023). They believe that negative supervisory feedback threatens their self-concept, self-ability, and social image belief, damaging their self-esteem level, and causing concern about their self-ability and self-social relationships. Employees also pursue security and stability in follow-up work to ensure the absence of mistakes or losses. Further, threat appraisal will lead to employee avoidance goal orientation, making them evade problems and task requirements (Higgins, 1997; Gutnick et al., 2012), pursue only security and stability needs, realize ought-self, and pay attention to the sense of loss at work. Consequently, negative performance indication's effect on negative supervisory feedback will likely work in self-focused and high-threat appraisal employees. Self-regulation through a defense mechanism's generation becomes a self-protection strategy for negative feedback receivers, benefiting their recovery from the pressure experience. In contrast, when the employee threat appraisal level is low, employees' attention and energy will be less focused on themselves because they will be less alert to external work situations. Therefore, negative supervisory feedback's negative performance indication effect is unlikely to be activated. The situation is no longer regarded as a threat to the self by employees, and the positive relationship between negative supervisory feedback and employee prevention motivation weakens. Previous studies have provided indirect evidence of threat appraisal's moderating effect. One study found that when threat appraisal was high, perceived creativity rewards were significantly negatively associated with creativity-related intrinsic motivation and creative performance (Li et al., 2017). Accordingly, we propose the following hypothesis:

H2: R&D employee threat appraisal positively moderates the relationship between negative supervisory feedback and prevention focus such that the positive relationship is stronger when threat appraisal is higher than when it is lower.

### 2.3 Prevention focus and employee creativity

Previous research has clearly signaled that prevention focus inhibits employee creativity's improvement. First, prevention-focused employees tend to adopt avoidant cognitive styles. Because of their emphasis on security and stability needs, they pursue the ought self and pay attention to negative outcomes. Prevention-focused employees usually adopt avoidance and vigilance cognitive strategies to prevent mistakes and risks. For example, they choose well-known and verified solutions to problems. Creativity refers to the generation of novel and useful ideas. It requires a flexible cognitive style to enhance this ability, that is, to encourage individuals to take risks and break the conventional methods of doing things to seek new solutions to problems (Baer et al., 2003). According to this logic, employee creativity decreases as prevention focus increases. Second, prevention focus affects the expansion of an individual's cognitive range. According to the creativity component model, domain-related knowledge, including knowledge and technology, is a component of creativity (Amabile, 1997). To generate novel and creative ideas, employees need perspectives and opinions from different sources to expand their knowledge range (Huang et al., 2014). The wider an individual's cognitive range, the easier it is to establish a connection between new cognitive elements, thus making it easier to generate novel and usable ideas. However, the prevention focus is primarily related to conducting analytical thinking, paying attention to accuracy and security, and insisting on local processing. Therefore, prevention-focused employees pay strong attention and maintain perseverance toward the cognitive elements initially retrieved, thus inhibiting their search for an increasing number of novel ideas. Supporting the above viewpoint, Lanaj et al. (2012)'s meta-analysis showed that prevention focus is negatively related to employee creativity. Through experimental and field studies, Kark et al. (2018) found that transactional leadership was positively associated with an employee prevention focus, reducing creativity. Accordingly, we propose the following hypothesis:

H3: R&D employee prevention focus negatively affects employee creativity.

### 2.4 Moderated mediation effect

The creativity component model proposes that task motivation (e.g., intrinsic and extrinsic motivation), domain-related knowledge, and creativity-related processes (e.g., individual cognitive models) are individual creativity's three key components. Leadership management practices influence creativity by influencing the above components (Amabile, 1997). Based on the above reasoning, we conclude that the interaction between negative supervisory feedback and employee cognitive appraisal indirectly affects creativity through prevention focus. The indirect effects are opposite in the two cognitive-appraisal conditions. When the employee challenge appraisal level is high, employees receiving negative supervisory feedback focus on the task level, produce problem-focused coping strategies, approach goal orientation, and strive to improve creativity and reduce their defensive motivation tendency. This helps them establish a broader thought-action system (Fredrickson and Losada, 2005) and improve their creativity by trying new ways to perform tasks. Therefore, when the employee challenge appraisal level is high, negative supervisory feedback is positively related to creativity through a prevention focus. Accordingly, we propose the following hypothesis:

H4: R&D employee challenge appraisal positively moderates the indirect effect of negative supervisory feedback on creativity through prevention focus such that the positive indirect relationship is stronger when challenge appraisal is higher than when it is lower.

However, when the employee threat appraisal level is high, employees receiving negative supervisory feedback focus on the self-level, become concerned about self-competence and self-social relationships, and develop emotion-focused coping strategies and avoidance goal orientation. This in turn strengthens their prevention motivation tendency. Prevention motivation narrows employee thought-action systems (Fredrickson and Losada, 2005) and makes them adopt existing task strategies to ensure daily tasks' safe and reliable execution. This is not conducive to developing creativity because creativity often needs to deviate from the norm and status quo (Anderson et al., 2014). Additionally, self-level goals compete with cognitive resources for task-level goals, affecting the realization of the latter, such as creative goals (Kluger and DeNisi, 1996). Therefore, when the employee threat appraisal level is high, negative supervisory feedback is negatively related to creativity through a prevention focus. Accordingly, we propose the following hypothesis:

H5: R&D employee threat appraisal negatively moderates negative supervisory feedback's indirect effect on creativity through prevention focus such that the negative indirect relationship is stronger when threat appraisal is higher than when it is lower.

## 3 Materials and methods

### 3.1 Participants and procedure

Data were collected using a questionnaire survey. Research samples were obtained from R&D employees of seven science and technology enterprises in Xi'an, Taiyuan, Nanjing, and other places in China, including from information transmission, chemical, machinery manufacturing, and other industries. R&D enterprises in science and technology enterprises often adopt the project team working method, with a direct supervisor leading each project team. In daily R&D work, R&D employees may receive both positive and negative feedback from supervisors on their output or behavior. At the same time, all of these industries encourage R&D employees to be creative by constantly optimizing technology to improve products' accuracy and reliability and offering new suggestions to complete work tasks. Our survey adopted the following procedures. First, we communicated with the survey unit's and the human resource department's senior managers to obtain their approval and support for the study. Second, the research team embedded itself deep in the survey unit to conduct on-the-spot collective measurements. Before the measurement, participants were informed about the notes and the process of answering the questions, as well as about the questionnaire survey's anonymity and confidentiality to ensure the questionnaire responses' quality. Finally, after the respondents completed the questionnaires, the results were coded immediately.

A total of 600 questionnaires were distributed, and 580 questionnaires were collected a response rate of 96.67%. According to the principle that all the items have the same option regularly or continuously checked, 67 invalid questionnaires were eliminated, leaving 513 valid questionnaires for an effective response rate of 88.45%. Of these participants, 40.2% were female; 30% were aged 20–30 years, 43.3% were aged 31–40 years, 19.3% were aged 41–50 years, and 7.4% were aged over 50 years; 7.2% had a college degree, 58.5% had a bachelor's degree, 32.4% had a master's degree, and 1.9% had a doctoral degree; and 29.4% had worked for 5 years or less, 22.8% had worked for 6–10 years, 19.1% had worked for 11–15 years, and 28.6% had worked for 16 years or more.

### 3.2 Measures

The measurement tools used in this study were all mature scales published in authoritative journals. We adopted a standard back-translation procedure adopted to ensure the consistency of content and meaning between the Chinese and English versions. First, members of the research team translated the scales into Chinese and then invited experts in the field with many years of experience studying abroad to translate it back to English, compare the differences before and after translation, and modify and improve the scale. All items used a 5-point Likert scale, ranging from 1 (“strongly disagree”) to 5 (“strongly agree”).

#### 3.2.1 Negative supervisory feedback

Employees evaluated negative supervisory feedback using a nine-item scale that Jaworski and Kohli (1991) developed, which was adapted for scientific and technological R&D contexts. The scale comprised two subscales: negative output feedback and negative behavioral feedback. One sample item wad as follows: “When my supervisor is upset by my work performance, he or she will tell me.” Cronbach's alpha of this scale was 0.848, the negative output dimension was 0.729, and the negative behavior dimension was 0.850.

#### 3.2.2 Challenge appraisal and threat appraisal

Employees reported their challenge and threat appraisal using an eight-item scale that Drach-Zahavy and Erez (2002) developed. The subscale had four items, and one sample item was “My job is a challenge to me.” The threat appraisal subscale had four items, and one sample item was “My job is a threat to me.” Cronbach's alpha of the challenge appraisal and threat appraisal subscales were 0.737 and 0.750, respectively.

#### 3.2.3 Prevention focus

Employees evaluated their prevention focus using a nine-item scale that Lockwood et al. (2002) developed. One sample item was as follows: “I am careful to avoid negative influence at work.” Cronbach's alpha for this scale was 0.777.

#### 3.2.4 Employee creativity

Employees reported their creativity using a 13-item scale George and Zhou (2001) developed. One sample item read, “I will propose new methods to achieve goals.” Cronbach's alpha for this scale was 0.934.

#### 3.2.5 Social desirability

Employees evaluated their social desirability using a five-item scale Andrews and Meyer (2003) developed. One sample item was as follows: “Sometimes I compare with others.” The Cronbach's alpha of this scale was 0.934.

### 3.3 Control variables

Because there may be individual differences (e.g., gender, age, education, and organizational tenure) in the interpretation of feedback information by feedback recipients (Kluger and DeNisi, 1996), we controlled for employee gender, education, and organizational tenure to avoid these unrelated variables' influence, confusing the causal relationship among the main variables in this study.

## 4 Results

### 4.1 Discriminant validity and common method bias testing

A confirmatory factor analysis was performed on the measured data using AMOS 24.0 to investigate the five main latent variables' discriminant validity. We compared the baseline model (five constructs: negative supervisory feedback, challenge appraisal, threat appraisal, prevention focus, and employee creativity) with five other models. If all measurement items were included in the observation indicators, the parameters to be estimated by the model would have exceeded the recommended ratio of the estimated parameters to the sample size (1:10) (Bentler and Chou, 1987). Therefore, the item packing method was adopted to estimate the latent variable constructs. The items with the highest and lowest loads were sequentially averaged using the item-to-construct balance method, and each one-dimensional construct's measurement items were packaged into two three-item groups. The analysis results (Table 1) showed that the fit index of the baseline model was good (χ^2^/df = 1.905, RMR = 0.015, root mean square error of approximation = 0.042, comparative fit index = 0.986 and normed fit index = 0.971, Tucker-Lewis index = 0.979, goodness of fit index = 0.974), and was significantly better than the others. This indicated that each core construct in this study had clear meaning and good discriminant validity.

**Table 1:** Results of the confirmatory factor analysis.

**Model**	**Factors**	**χ^2^/df**	**RMR**	**RMSEA**	**CFI**	**NFI**	**TLI**	**GFI**
6-factor model	NF; CA; TA; PF; EC; CMV	2.150	0.015	0.047	0.980	0.964	0.967	0.969
5-factor model	NF; CA; TA; PF; EC	1.905	0.015	0.042	0.986	0.971	0.979	0.974
4-factor model	NF+CA; TA; PF; EC	5.043	0.023	0.089	0.931	0.916	0.906	0.935
4-factor model	NF+TA; CA; PF; EC	5.215	0.024	0.091	0.928	0.914	0.902	0.932
3-factor model	NF+CA+TA; PF; EC	9.403	0.040	0.128	0.849	0.834	0.804	0.873
2-factor model	NF+CA+TA+PF; EC	13.939	0.059	0.159	0.758	0.745	0.698	0.812
1-factor model	NF+CA+TA+PF+EC	22.699	0.069	0.206	0.586	0.577	0.494	0.713

*N* = 513. NF, negative feedback; CA, challenge appraisal; TA, threat appraisal; PF, prevention focus; EC, employee creativity; CMV, common method variance (social desirability).

Common method bias is prone to occur when multiple variables are collected using a single-source self-report method, thus reducing research validity (Podsakoff et al., 2003). Following Zhou and Long (2004), procedural and statistical controls were adopted to reduce its impact. Regarding procedural control, first, an anonymous filling method was used to allow respondents to answer truthfully and to ensure scientific and reliable feedback acquisition. Data confidentiality and the use of data for only academic research purposes were emphasized. Second, all measurement items were designed to avoid semantic ambiguity and reveal subjective attitude tendencies; specific cognitive and behavioral reports were used instead for items social desirability bias had obviously influenced. Third, two sets of research questionnaires were designed to disrupt the overall questionnaire's arrangement order and the research variables' internal items to avoid the single thinking by respondents. Regarding statistical control, Harman's single-factor test and single method-factor approach were used to test common method bias. The test results showed that fit indices of single-factor models were not ideal (χ^2^/df = 22.699, RMR = 0.069, root mean square error of approximation = 0.206, comparative fit index = 0.586, normed fit index = 0.577, Tucker-Lewis index = 0.494, goodness of fit index = 0.713). This indicated that common method bias had little effect on this study's results. Because the constructs in this study mainly involve negative attitude evaluations, social desirability may be the primary source of common method bias (Podsakoff et al., 2003). Therefore, the single method-factor approach was used for retesting. After adding a method factor (i.e., social desirability) to the baseline model, the six-factor model's fit indices became significantly worse, Δχ^2^_(11)_ = 34.451, *p* < 0.001. As a result, there were no significant common method biases among the study variables.

### 4.2 Descriptive statistics and correlation analysis

Table 2 reports the descriptive statistics and correlations for the study variables. Bivariate correlations showed that, negative supervisory feedback was positively related to prevention focus but was not significant (r = 0.013, *p* > 0.05), and it was negatively related to employee creativity but was not significant (r = −0.014, *p* > 0.05). Prevention focus is negatively associated with employee creativity (r = −0.102, *p* < 0.05). Challenge appraisal is negatively associated with prevention focus but not significantly so (r = −0.007, *p* > 0.05), and it is positively associated with employee creativity (r = 0.284, *p* < 0.01). Threat appraisal is positively associated with prevention focus (r = 0.306, *p* < 0.01) and negatively associated with employee creativity (r = −0.288, *p* < 0.01). These findings provided preliminary support for our hypotheses.

**Table 2:** Means, standard deviations, and correlation coefficients of the study variables.

**Variable**	**M**	**SD**	**1**	**2**	**3**	**4**	**5**	**6**	**7**	**8**
1. Gender	1.402	0.491	—							
2. Education	2.290	0.625	0.007	—						
3. Organizational tenure	3.532	1.608	−0.029	−0.349^**^	—					
4. Negative supervisory feedback	3.416	0.482	−0.084	0.013	−0.159^**^	—				
5. Challenge appraisal	3.438	0.587	−0.058	0.094^*^	−0.022	0.088^*^	—			
6. Threat appraisal	2.333	0.681	−0.023	−0.088^*^	−0.074	0.021	−0.132^**^	—		
7. Prevention focus	3.207	0.511	−0.098^*^	−0.026	−0.009	0.013	−0.007	0.306^**^	—	
8. Employee creativity	3.518	0.521	−0.105^*^	0.040	0.163^**^	−0.014	0.284^**^	−0.288^**^	−0.102^*^	—

^*^p < 0.05, ^**^p < 0.01.

### 4.3 Hypothesis testing

We examined our hypotheses in SPSS 26.0 using hierarchical regression analysis and conditional indirect effect analysis, with gender, educational background, and organizational tenure as control variables. We followed the process Edwards and Lambert (2007) recommended to test the moderated mediation effect. We first tested the moderating effect on the mediating variable, then we examined the predictive effect of the mediating variable on the dependent variable, and finally we tested the moderated mediation effect in the first stage. Even though some researchers have argued that it is theoretically necessary to discuss the mediating effect between independent and dependent variables in terms of statistics, it is not essential to test it. This average mediating effect may not be significant because of the potential existence of moderate variables (Chen et al., 2012). To avoid multicollinearity, we centered on the variables used to form the interaction terms before hypothesis testing. Table 3 summarizes the regression results. because of cognitive appraisal's potential presence as a moderating variable, negative supervisory feedback was not associated with either prevention focus (β = 0.001, *p* > 0.05) or employee creativity (β = 0.008, *p* > 0.05). Different types of cognitive appraisal changed the direction of negative leadership feedback's effect on prevention focus and creativity rather than simply enhancing or weakening their relationship. This was consistent with our theoretical hypothesis.

**Table 3:** Results of the hierarchical regression analysis.

**Variable**	**Prevention focus**						**Employee creativity**		
	**Model 1**	**Model 2**	**Model 3**	**Model 4**	**Model 5**	**Model 6**	**Model 7**	**Model 8**	**Model 9**
**Controls**									
Gender	−0.099^*^	−0.099^*^	−0.099^*^	−0.102^*^	−0.091^*^	−0.092^*^	−0.100^*^	−0.100^*^	−0.111^*^
Education	−0.033	−0.033	−0.032	−0.024	0.007	0.009	0.110^*^	0.111^*^	0.107^*^
Organizational tenure	−0.023	−0.023	−0.023	−0.010	0.014	0.016	0.199^***^	0.200^***^	0.198^***^
**Independent variable**									
Negative supervisory feedback		0.001	0.002	0.007	0.001	0.012		0.008	0.008
**Moderators**									
Challenge appraisal			−0.010	−0.001					
Threat appraisal					0.305^***^	0.283^***^			
Negative supervisory feedback × Challenge appraisal				−0.139^**^					
Negative supervisory feedback × Threat appraisal						0.124^**^			
**Mediator**									
Prevention focus									−0.108^*^
R^2^	0.011	0.011	0.011	0.030	0.102	0.117	0.047	0.048	0.059
ΔR^2^	0.011	0.000	0.000	0.019	0.091	0.015	0.047	0.000	0.012
F	1.850	1.385	1.116	2.601^*^	11.504^***^	11.134^***^	8.456^***^	6.338^***^	6.368^***^

*N* = 513. ^*^p < 0.05, ^**^p < 0.01, ^***^p < 0.001. All coefficients were standardized.

**Figure 2:**
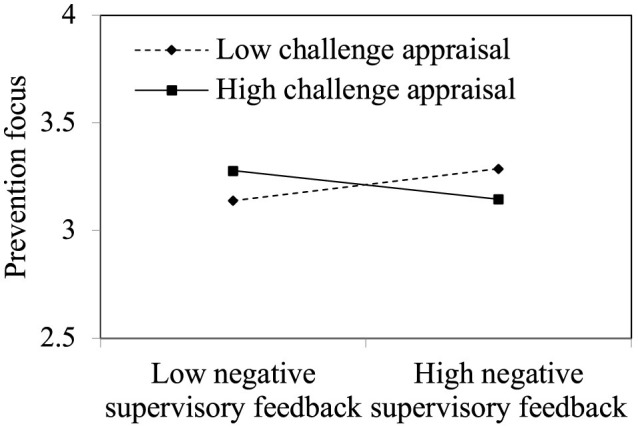
Moderating effect of challenge appraisal.

H1 proposed that challenge appraisal negatively moderates negative supervisory feedback's effect on prevention focus, such that the positive relationship is weaker when challenge appraisal is higher than when it is lower. Consistent with this hypothesis, the interaction term for negative supervisory feedback and challenge appraisal was negatively related to prevention focus (Model 4: β = −0.139, *p* < 0.01). We plotted the relationships between negative supervisory feedback and prevention focus at high and low challenge appraisal levels (one standard deviation above and below the mean). In Figure 2, simple slope tests showed that negative supervisory feedback was negatively related to prevention focus for individuals with high challenge appraisal (simple slope β = −0.216, t = −2.529, *p* < 0.05) and positively associated with prevention focus for individuals with low challenge appraisal (simple slope β = 0.250, t = 2.860, *p* < 0.01). Thus, H1 was supported.

**Figure 3:**
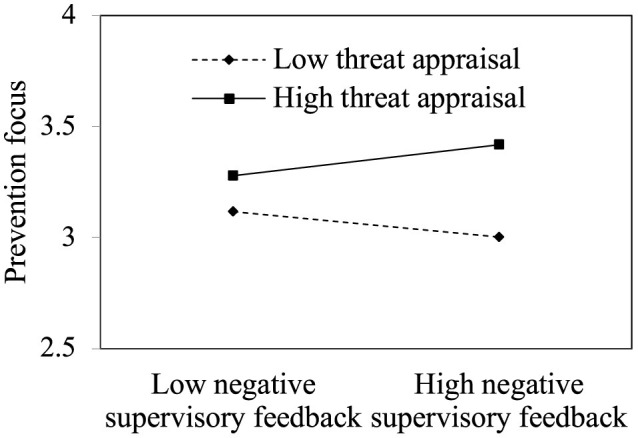
Moderating effect of threat appraisal.

H2 proposed that threat appraisal positively moderates negative supervisory feedback's effect on prevention focus, such that the positive relationship is stronger when threat appraisal is higher than when it is lower. The interaction terms for negative supervisory feedback and threat appraisal were positively related to prevention focus (Model 6: β = 0.124, *p* < 0.01). We also plotted the relationships between negative supervisory feedback and prevention focus at high and low levels of threat appraisal (one standard deviation above and below the mean). As Figure 3 shows, simple slope tests showed that negative supervisory feedback was positively related to prevention focus for individuals with high threat appraisal (simple slope β = 0.197, t = 2.513, *p* < 0.05) and negatively associated with prevention focus for individuals with low threat appraisal (simple slope β = −0.163, t = −2.256, *p* < 0.05). Thus, H2 was supported.

H3 suggested that prevention focus was negatively related to employee creativity. Model 9 in Table 3 shows that R&D employee prevention focus was negatively and significantly associated with employees' creativity (β = −0.108, *p* < 0.05), lending support to H3.

Hypotheses 4 and 5 posited a conditional indirect effect model whereby the interactive effects of negative supervisory feedback and challenge or threat appraisal would influence employee creativity through its effect on prevention focus. We examined this model using Hayes (2013) PROCESS macro to bootstrap 5,000 samples and estimate the bias-corrected 95% confidence intervals (CIs). The results in Table 4 support the conditional indirect effect. For challenge appraisal, negative supervisory feedback's indirect effect on employee creativity via prevention focus was positive for individuals with high challenge appraisal (indirect effect value = 0.015, 95% CI = [0.001, 0.049], excluding zero) and negative for individuals with low challenge appraisal (indirect effect value = −0.017, 95% CI = [−0.049, −0.002], excluding zero). The moderated mediation effect index was significant (index = 0.028, 95% CI = [0.004–0.070], excluding zero). These results supported H4.

**Table 4:** Bootstrap test results of moderated mediation effect.

**Moderating variable**	**Indirect effect**	**SE**	**Indirect effect 95% CI**
Challenge appraisal as a moderator	Negative supervisory feedback → Prevention focus → Employee creativity		
High challenge appraisal (+1 SD)	0.015	0.011	[0.001, 0.049]
Low challenge appraisal (−1 SD)	−0.017	0.011	[−0.049, −0.002]
Moderated mediation effect index	0.028	0.016	[0.004, 0.070]
Threat appraisal as a moderator	Negative supervisory feedback → Prevention focus → Employee creativity		
High threat appraisal (+1 SD)	−0.016	0.010	[−0.044, −0.003]
Low threat appraisal (−1 SD)	0.013	0.010	[0.001, 0.042]
Moderated mediation effect index	−0.021	0.012	[−0.055, −0.004]

For threat appraisal, negative supervisory feedback's indirect effect on employee creativity via prevention focus was negative for individuals with high threat appraisal (indirect effect value = −0.016, 95% CI = [−0.044, −0.003], excluding zero), and positive for individuals with low threat appraisal (indirect effect value = 0.013, 95% CI = [0.001, 0.042], excluding zero). The moderated mediation effect index was significant (index = −0.021, 95% CI = [−0.055, −0.004], excluding zero). These results supported H5.

## 5 Discussion

Our main research objective was to resolve theoretical and empirical inconsistencies in the relationship between negative supervisory feedback and employee creativity. The results of our field study demonstrated that the relationship between these factors depends on the recipient's cognitive appraisal. We found that negative supervisory feedback was negatively associated with prevention focus for individuals with high challenge appraisal and low threat appraisal. In contrast, it was positively associated with prevention focus for individuals with low challenge appraisal and high threat appraisal. R&D employee prevention focus was negatively related to creativity. Our moderated mediation results revealed distal consequences of cognitive appraisal's moderation. Negative supervisory feedback's indirect effect on employee creativity via prevention focus was positive for high challenge appraisal and low threat appraisal individuals and negative for low challenge appraisal and high threat appraisal individuals.

### 5.1 Theoretical contributions

Our study contributes to the creativity and feedback literature. First, we integrated the contradictory findings on the relationship between negative feedback and creativity. By reviewing previous literature, we found that the current evidence on the relationship between negative feedback and creativity is contradictory. For example, some researchers have argued that negative feedback will cause employees to feel insecure or threatened, diverting their attention from creative tasks and focusing it on the self-level, thereby hindering the improvement of creativity (Van Dijk and Kluger, 2011). Other researchers have suggested that negative feedback creates a gap in creativity standards, which makes them focus on the task level and on constant gap improvement, thereby contributing to creativity improvement (Hoever et al., 2018). Furthermore, promoting creativity through feedback is not easy and intuitive, and it is essential to examine feedback's, feedback recipient attributes', and feedback provider attributes' nature and composition to increase feedback strategies' effectiveness for stimulating employee creativity (Zhou, 2008). Our research introduced feedback recipient attributes (individual cognitive appraisal) as the moderating variables, integrating contradictory findings on the relationship between negative feedback and creativity into a coherent theoretical model, and investigated how individual differences in feedback recipients resolve this inconsistency. We provided a comprehensive and concise theoretical framework integrating the two seemingly opposing views and responding to the call to examine the boundary conditions between feedback and creativity, thus contributing to the research on creativity and feedback.

Second, our study expanded the boundary conditions of the association between negative supervisory feedback and motivation. Although previous studies have linked negative supervisory feedback with prevention focus (Li et al., 2013), they have rarely paid attention to the contingency of the relationship between them. By combining feedback intervention and cognitive appraisal of stress theories, we examined the conditions under which negative supervisory feedback strengthens or weakens recipient prevention focus. The interaction effect's results revealed that employees who receive negative supervisory feedback adjust their motivation strategies according to the two cognitive appraisal styles. This is consistent with a previous research conclusion that negative feedback and individual differences interact, influencing individual behavioral consequences (Holderness et al., 2016). The findings also expand the viewpoint of feedback intervention theory, which indicates feedback recipients' personal attributes determine whether they focus their attention on the task-level or self-level, subsequently associated with positive or negative individual consequences respectively (Kluger and DeNisi, 1996). However, this theory does not indicate when individuals have task- or self-orientation in response to negative feedback. Our research proposed an essential but neglected boundary condition from feedback recipient attributes' perspective, enhancing the understanding of when there is a positive or negative correlation between negative supervisory feedback and motivational consequences (e.g., prevention motivation) and thus contributing to negative feedback valence research.

Third, this investigation enriched the research on the mediating mechanism of the relationship between negative supervisory feedback and creativity. Integrating the literature on negative supervisory feedback, prevention focus, and creativity, we proposed a moderated mediating effect model, emphasizing that prevention focus is a vital motivation mechanism in the relationship between negative supervisory feedback and creativity. Previous studies have also shown that negative feedback can promote or inhibit individual performance outcomes depending on the situation (Holderness et al., 2016). However, few studies have explicitly discussed the mediating mechanism of the interaction effect between negative feedback and situational factors. To this extent, we expanded the research field by verifying prevention focus's critical role in the interaction between negative feedback and cognitive evaluation. The moderated mediating results also enhanced our understanding of the mediation mechanism between feedback valence and creativity. Previous studies on the relationship between feedback and creativity have been primarily based on the intrinsic motivation framework, and it is believed that negative feedback affects creativity by influencing employee intrinsic motivation. However, because regulatory focus is considered a motivation-based individual trait or state, compared to other individual constructs, it emphasizes on individual action strategies and their effect on goal achievement, making its influence on behavior more direct (Gamache et al., 2015). Therefore, from the regulatory focus perspective, this study revealed the mediating effect of prevention focus on the interaction between feedback valence and situational factors on creativity, providing a unique and novel theoretical explanation.

### 5.2 Practical implications

Our research offers important implications for managerial practices. First, we encourage managers to adjust their feedback strategies reasonably. Negative supervisory feedback can promote or inhibit prevention focus and creativity depending on the feedback recipients' attributes. Therefore, managers should reasonably adjust their strategies when using feedback tools. It is necessary to consider the effect of combining negative feedback with individual cognitive appraisal. For individuals with high challenge appraisal, giving negative feedback appropriately enables them to find potential growth and development opportunities and improve the creativity gap. For individuals with a high threat appraisal, negative feedback should be avoided as much as possible to reduce the possibility of perceived harm or loss. Instead, negative feedback can be provided to them at the end of the creative task, so that they have enough time to manage the perceived threat.

Second, considering that R&D employees' cognitive appraisal styles change motivation and behavioral patterns' direction, managers should pay sufficient attention to it. Supervisors can identify and differentiate R&D employees' cognitive appraisal methods by observing their daily work behaviors, seeking feedback on leadership behaviors, and then giving them appropriate positive or negative feedback. Furthermore, supervisors should also utilize the incentive function of feedback tools, emphasizing feedback's potential benefits for R&D work, and encouraging R&D employees to respond to external feedback actively. For example, cultivating self-efficacy and setting examples promote challenge appraisal.

Finally, managers should work on scientifically stimulating R&D employees' regulatory focus. Our findings suggest that R&D employees' prevention focus is negatively related to their creativity. Managers should make efforts to stimulate their promotion focus and guide their prevention focus. Specifically, supervisors can stimulate employee promotion focus by emphasizing risk-taking and flexibility, encouraging optimism and new working methods, and fostering an open team culture. Meanwhile, individuals with a high prevention focus should be encouraged to put forward different suggestions or novel ideas, and the incentive mechanism should be improved to reduce the risks of their innovative behavior and increase returns accordingly. These would give full play to their innovation enthusiasm and initiative.

### 5.3 Limitations and future research

Despite its contributions, our study has some limitations that should be addressed in future research. First, a cross-sectional design was used to test the hypotheses. The data for all research variables were collected at the same point of time, ignoring the time effect's influence on the research results and making it challenging to examine the causal relationship among variables accurately. A long-term research design of involving time series can be considered in the future to increase the research conclusions' explanatory power. Second, we selected the control variables. Although the present study controlled for demographic information's influence on the relationship among research variables, individuals' feedback-seeking behavior and learning goal orientation—representing their willingness to accept negative feedback as well as their motivation to acquire new skills and new development—may affect the relationship between negative supervisory feedback and creativity. Therefore, these should be controlled in future research. Thirdly, we focused on individual cognitive appraisal attribute as critical contingency factors that influence employees' regulatory focus of perceived negative supervisory feedback. However, supervisory feedback style factors may also have similar effects. For example, supervisory challenge and threat feedback styles may trigger different employee appraisal styles, thereby strengthening or weakening the incentive effect of perceived negative feedback. Future research can explore this issue, which can shed further light on what can influence employees' motivational mechanisms ensuing from perceived negative feedback. Finally, our study examined only the double-edged sword effect of negative feedback. However, it is still unclear whether positive feedback can promote or inhibit creativity, and whether regulatory focus theory can explain the above inconsistent relationship. Future research should include positive feedback in the research model to investigate common influences and differences between the two feedback forms and to comprehensively reveal the relationship between feedback valence and creativity.

## Data availability statement

The raw data supporting the conclusions of this article will be made available by the authors, without undue reservation.

## Ethics statement

The studies involving humans were approved by the School of Humanities and Social Science, Beijing Institute of Technology, Beijing, China. The studies were conducted in accordance with the local legislation and institutional requirements. The participants provided their written informed consent to participate in this study. Written informed consent was obtained from the individual(s) for the publication of any potentially identifiable images or data included in this article.

## Author contributions

HL: Writing – review & editing, Writing – original draft, Software, Methodology, Investigation, Data curation, Conceptualization. JZ: Writing – review & editing, Writing – original draft, Project administration, Methodology, Investigation, Formal analysis, Data curation, Conceptualization. MB: Writing – review & editing, Writing – original draft, Methodology, Conceptualization. ME: Writing – review & editing, Writing – original draft, Methodology, Conceptualization. JZ: Writing – review & editing, Writing – original draft, Visualization, Validation, Software, Resources, Formal analysis. XX: Writing – review & editing, Writing – original draft.
